# Intestinal Fistulæ

**Published:** 1853-04

**Authors:** De Laskie Miller

**Affiliations:** Chicago, Ill.


ART. II.—*Report of Two Cases of Intestinal Fistulæ.* By DE LASKIE MILLER, M. D., Chicago, Ill.

CASE 1.—Visited——Johnson, aged about 12 years, January 22nd, 1850, found him with considerable febrile movement, with severe pain in the right iliac region, increased on pressure, and continuous, bowels constipated. Directed fomentations to the painful part and ordered a Cathartic, to be followed by a full dose of Dover Powder. 23rd.—The Cathartic operated kindly, removing a large quantity of hardened fæces. His pulse is 120 in the minute—tonge dry—pain in the right iliac region, about the same as yesterday. Some tumefaction corresponding to the seat of pain.—Continue formentations, and prescribed the following:—℞ Hyd. Prot Chlor. grs.viij. Pulv. Doveri ℈j.—M. Div. Chart No. 4. take one every fourth hour. 24th.—Patient, much the same as yesterday—pulse rather softer and 110—tumefaction increasing, but circumscribed. Ordered a laxative, to be followed by Dover Powder, sufficient to quiet the pain. Applied a blister to the whole extent of the painful part.

The blister drew well, and discharged freely, the tongue became moist—the pain gradually subsided, though tenderness to pressure remained—the swelling increases, without extending its boundary, which is about five inches in diameter. After several days, detect fluctuation in the part, there seems to be great exhaustion of the vital forces, the patient complains of debility, and a sense of depression. He states, that two days before I visited him, he received an injury at the point affected, by falling upon a billet of wood.

Punctured the swelling, which discharged a large quantity of fœtid gas, and a small quantity of grumous blood, mixed with unhealthy pus—the tumefaction immediately subsided, and a distinct ring marking the boundary of the swelling remained. Ordered a cataplasm to be applied to the part, and prescribed :—Sulph. Morphia, as occasion required, to keep the patient quiet. February 10th.—Three days after the abscess was punctured, the discharge consisted almost entirely of fæces, with portions of partly digested food; no natural evacuation from the bowels during the last three days. Ordered injections.

The anema produced a slight evacuation from the bowels; the patient feels but little pain. Advised a nourishing diet, and porter. March 15th.—The patient improving in strength—the Artificial opening assuming the appearance of a fistulous orifice, and discharges as last noted. The lower portion of the bowels have been kept open by the use of injections, alternated with croton oil. April 15th.—The patient so much improved, as to ride 25 miles, to the residence of his father. The discharge the same in quality as last noted, but less in quantity. The orifice is evidently smaller. January.—One year after the attack, the patient has perfectly recovered; the orifice continued to contract, the discharge becoming less, and finally ceased altogether.

CASE 2.—Niles had been sick two weeks, and under treatment for inflammation of the bowels, when I was called to see him, in consultation, found him with pain in the right iliac region, which was increased on pressure, had taken Calomel largely, and been catharticised freely. A blister was applied over the seat of the pain, and anodynes prescribed. About two weeks subsequent to this time, I was requested to visit him again, and take charge of the case, he was at this time very much debilitated, pain and tenderness in the iliac region same as before, but found an abscess pointing about two inches to the right side of the spinous process of the second lumbar vertebra: punctured it, when it discharged freely, pus intermingled with flakes of lymph, poultices were applied to the part, and tonics prescribed. On the second day after the abscess was opened, fæces were observed to escape from the orifice, and occasionally portions of undigested food. About three weeks subsequent to this, another opening was formed about two inches internal to the anterior superior spinous process of the ilium, giving exit to a similar discharge. The opening on the back now began to contract, and finally closed, about six weeks after it was formed. After the time the first opening was made, it was difficult to produce an evacuation per anum. Injections were used, alternated with croton oil.

This course was followed for near six months, and the patient sustained by tonics and a nourishing diet, when the fistulous opening gradually contracted, occasionally closing for a few days, then opening for a short time, and discharging as before, till at the end of eighteen months, when it remained closed, and the patient regained his usual strength and spirits.

*Remarks.*—There seems but little doubt, that in the case of Johnson, the inflammatory action, and consequent ulceration were induced by the injury received by the fall, the billet of wood forcing the walls of the abdomen, and coats of the intestine, against the accumulation of hardened fæces, contained within the Colon at the time, producing contusion, and possibly slight laceration of the coats of the intestine. We see exemplified in this case the beautiful provision of nature, which by forming adhesions between the various structures, around the injury, prevents the fatal consequences that would ensue, from the escape of foreign substances into the peritoneum. A point of practical importance is the propriety of the early openings, from the great depression of the vital forces; the inference in this case was, that the contents of the abscess were producing serious mischief, which was manifested upon the escape of the fœtid gas, and the relief which followed.

In the case of Niles, is it not probable that some solid foreign body had found its way, perhaps, into the appendix vermiformis, whence it could not dislodge itself, causing the inflammation and ulceration, and leading to the abscess? None such was discovered in the discharges, although the attention of the parents was directed to it. But it is easy to understand how it might escape unobserved in the great amount of matter that was constantly discharging. Not the least practical point in these cases is the use of croton oil, this article fulfilled the indicatives admirably, it produced a uniform peristaltic action throughout the entire extent of the intestinal canals, without irritating the internal orifice of the fistula, by its bulk. In the management of the above cases, the reader will notice an apparent disregard of the opinions of the older physicians, "who, in injuries of the abdomen, extending to the intestines, never left anything to nature."

